# Novel Biochemical Findings from *Bothrops marajoensis* Venom

**DOI:** 10.3390/ijms27198524

**Published:** 2026-09-24

**Authors:** Isadora Oliveira, Isabela Ferreira, Beatriz Jacob, Emilly Ramos, Lorena Agati, Isadora Lino, Francielle Cordeiro, Felipe Costa, Eloise Filardi, Manuela Pucca, Eliane Arantes

**Affiliations:** 1 Institute of Agricultural, Exact and Biological Sciences of Iturama, Federal University of Triângulo Mineiro, Iturama 38280-000, Minas Gerais, Brazil; isadora_so@yahoo.com; 2 Department of BioMolecular Sciences, School of Pharmaceutical Sciences of Ribeirão Preto, University of São Paulo, Ribeirão Preto 19040-903, São Paulo, Brazil; igobboferreira@alumni.usp.br (I.F.); beatrizjacobcs@gmail.com (B.J.); emilly.ramos@usp.br (E.R.); lorenaagati06@usp.br (L.A.); isadora.lino@usp.br (I.L.); fran_ac@usp.br (F.C.); 3 Department of Clinical Analysis, School of Pharmaceutical Sciences, São Paulo State University, Araraquara 19060-900, São Paulo, Brazil; felipe.costa08@unesp.br (F.C.); e.filardi@unesp.br (E.F.); manuela.pucca@unesp.br (M.P.)

**Keywords:** *Bothrops marajoensis*, snake venom, *Bothrops*, antivenom, phosphodiesterase, myotoxicity, C2C12 cells

## Abstract

*Bothrops marajoensis* is a Brazilian pit viper whose venom remains poorly characterized compared with those of other medically important *Bothrops* species. In the present study, we performed a comparative biochemical characterization of *B. marajoensis* venom with six additional *Bothrops* venoms (*B. alternatus*, *B. atrox*, *B. jararaca*, *B. jararacussu*, *B. moojeni*, and *B. neuwiedi*). Venoms were analyzed by reversed-phase chromatography, electrophoresis, immunorecognition by commercial bothropic antivenom, phosphodiesterase activity, and their effects on the viability of differentiated C2C12 myotubes. Chromatographic and electrophoretic analyses revealed a largely conserved overall venom composition among species, with qualitative differences, although quantitative differences in protein relative abundance were evident. Bothropic antivenom recognized all venoms, indicating broad antigenic cross-reactivity despite venom variation. *B. marajoensis* exhibited intermediate phosphodiesterase (PDE) activity relative to other species evaluated, confirming functional variability among *Bothrops* venoms. In the cell viability assay, *B. marajoensis* displayed comparatively low apparent cytotoxicity toward differentiated C2C12 myotubes, maintaining higher cell viability than highly myotoxic venoms such as those of *B. jararacussu* and *B. moojeni*. These findings expand the biochemical characterization of *B. marajoensis* venom and demonstrate that, although venoms share the conserved toxin repertoire typical for *Bothrops* genus, quantitative differences influence its biological activities, preserving broad recognition by bothropic antivenom.

## 1. Introduction

Snake venoms are complex mixtures of bioactive molecules that have evolved to immobilize prey and deter predators, often causing profound physiological effects on various systems [1,2]. Among the diverse group of venomous snakes, *Bothrops marajoensis*—a species endemic to Marajó Island in Brazil—has attracted growing scientific interest because of the unique composition and multifaceted biological actions of its venom [1].

Studies have revealed that *B. marajoensis* venom exhibits a wide range of pharmacological activities, including neurotoxic, myotoxic, cytolytic, cardiotoxic, nephrotoxic, antibacterial, and antiparasitic effects [3,4,5]. A case report of a male child (<10 years old) from Marajó Island, Brazil, shows a severe *B. marajoensis* envenomation to his right foot, with transport complications that delayed medical care by 25 h, during which the patient developed somnolence and a comatose state. Upon hospital admission, he presented extensive right lower-limb edema, ecchymosis, hematuria, and severe neurological impairment. Cranial computed tomography revealed a right frontal intracerebral hemorrhage, manifesting clinically with right-sided flaccid hemiplegia and leftward labial commissure deviation. Treatment included 12 vials of bothropic antivenom, conservative clinical management, and physical therapy. Following 19 days of hospitalization, the patient was discharged with permanent right flaccid hemiplegia, representing the first documented case of pediatric hemorrhagic stroke caused by *B. marajoensis* envenomation [6].

Regarding the venom’s biological functions, a study investigated the impact of *B. marajoensis* venom, focusing on its neurotoxic and myotoxic effects in isolated neuromuscular preparations from chick biventer cervicis (CBC) and mouse phrenic nerve-diaphragm (PND). The venom induces dose-dependent neuromuscular blockade: at lower doses (1 μg/mL in CBC and 5 μg/mL in PND), it inhibits neuromuscular activity without harming muscle tissue; at higher doses (20 μg/mL in PND), it causes both neuromuscular blockade and moderate muscle damage. These findings indicate that *B. marajoensis* venom can impair neuromuscular function, with blockade often leading to muscle edema [1].

To date, only a limited number of toxins have been isolated from *B. marajoensis* venom, including a thrombin-like serine protease (TLBm), Lys49 phospholipase A_2_ (PLA_2_) homologs, a metalloproteinase, and an L-amino acid oxidase [7,8,9,10]. Examples of Lys49 PLA_2_ are the BmjeTX-I and BmjeTX-II isoforms from *B. marajoensis* venom, which were characterized by biochemical and mass spectrometric approaches. Both are basic PLA_2_ myotoxins highly homologous to those found in other *Bothrops* species. Ex vivo, they blocked neuromuscular transmission, suggesting a presynaptic mechanism. In mice, both induced myonecrosis, increased systemic IL-6 levels, and caused moderate paw edema, indicating local vascular permeability [8].

In addition to their myotoxic effects, *B. marajoensis* toxins can interfere with hemostatic processes. The thrombin-like serine protease, TLBm was purified from *B. marajoensis* venom and characterized as a 33.3 kDa single-chain protein with a conserved catalytic triad. Functionally, TLBm acts as an α,β-fibrinogenase and induces serine protease-dependent platelet aggregation. Despite causing minimal local edema, its procoagulant activity may contribute to systemic hemostatic disturbances during envenomation [10].

Hemostatic alterations are also associated with the snake venom metalloproteinase (SVMP) component of the venom. BmHF-1 is a P-I class SVMP isolated, with 27.2 kDa containing the conserved zinc-binding motif and Met-turn characteristic of metzincins. BmHF-1 preferentially hydrolyzes the Aα-chain of fibrinogen and is inhibited by metal chelators, confirming its SVMP activity. Functionally, it induces weak hemorrhage and dose-dependent local edema, but no myotoxicity, suggesting a predominant role in local tissue inflammation during envenomation [9].

Beyond myotoxic, inflammatory, and hemostatic effects, *B. marajoensis* venom also contains toxins with potential antimicrobial and antiparasitic activities. BmarLAAO is a 67–72 kDa acidic L-amino acid oxidase (LAAO) purified from *B. marajoensis* venom. It exhibits dose-dependent antimicrobial activity against Gram-positive and Gram-negative bacteria and potent leishmanicidal effects against *Leishmania* spp. Notably, BmarLAAO shows low cytotoxicity toward murine macrophages, highlighting its potential as a natural lead for antimicrobial and antiparasitic drug development [11]. This highlights the still fragmentary understanding of its venom composition and its contribution to the observed toxicological and therapeutic properties. Given the emerging evidence of the venom’s diverse bioactivities and the identification of novel toxins with potential pharmacological value, this article aims to provide a comprehensive overview of the biochemical and biological properties of *B. marajoensis* venom, seeking to elucidate the molecular underpinnings of its toxic effects and to explore prospects for therapeutic development based on its unique constituents.

## 2. Results

### 2.1. Venom Fractionation and Comparision

Venom fractionation from seven *Bothrops* species (*B. alternatus*, *B. atrox*, *B. jararaca*, *B. jararacussu*, *B. marajoensis*, *B. moojeni*, and *B. neuwiedi*) is presented in Figure 1A–G. All venoms exhibited complex and heterogeneous elution patterns, with multiple peaks distributed across the chromatographic gradient, consistent with the known biochemical complexity of *Bothrops* spp. venoms. A conserved overall profile was observed across species, with components eluting in early, intermediate, and late elution volumes. Early eluting fractions likely correspond to more hydrophilic and/or low-molecular-weight components, whereas late-eluting peaks suggest the presence of more hydrophobic and larger toxins. However, significant interspecific variation was evident in both peak distribution and relative abundance. For example, *B. jararacussu* and *B. moojeni* showed higher peaks in intermediate elution profile. As observed in Figure 1D,F, fraction 6 was more abundant and evident in these species.

These similarities and differences in venom composition are evident when we overlay the chromatograms in Figure 2. The intermediate peak is even more apparent in the venoms of *B. jararacussu*, *B. moojeni*, and *B. atrox*, although the retention time differs slightly for the latter. A notable feature was observed in *B. jararacussu*, whose chromatogram showed a dominant peak in the middle region of the profile, in contrast to the more evenly distributed peaks in the other species.

The electrophoretic profiles obtained by 13.5% SDS-PAGE of *Bothrops* spp. venoms indicate the presence of distinct components with different molecular masses (Figure 3A,B). Under non-reducing conditions, the electrophoretic profile showed a prominent band in the 50–60 kDa range in the venoms of *B. alternatus*, *B. atrox*, and *B. jararaca*, suggesting the presence of SVMPs. However, differences in band intensity among species suggest quantitative variation in SVMPs subclasses rather than the presence of unique proteins among these species. In contrast, the venoms of *B. jararacussu*, *B. neuwiedi*, *B. moojeni*, and *B. marajoensis* exhibited distinct electrophoretic profiles, differing in both their banding patterns and the distribution of proteins according to molecular weight (Figure 3A). Under reducing conditions, evident changes were observed, particularly for *B. moojeni* and *B. marajoensis*, whose banding patterns became more similar under these conditions (Figure 3B).

Tricine SDS-PAGE provides improved resolution of low-molecular-weight components present in the venom samples. Thus, under non-reducing conditions, a prominent band around 20 kDa was observed in all species (Figure 3C). Nevertheless, *B. jararacussu*, *B. moojeni* and *B. neuwiedi* venoms exhibited more pattern similarity between 14.2–17 kDa range. Thus, it corroborates with the pronounced intermediate peaks presented in chromatographic analysis. This range indicates the existence of PLA_2_ myotoxins, usually found between the 14–20 kDa. Furthermore, these species showed considerable similarity, particularly due to the prominent bands at approximately 26, and 45 kDa. Conversely, the more evenly distributed chromatographic profiles of *B. marajoensis* and *B. alternatus* suggest lower relative PLA_2_ abundance. In addition, the similarity among the electrophoretic profiles of *B. alternatus*, *B. atrox*, and *B. jararaca* remained evident, reinforcing, above all, the SVMP presence, whereas *B. marajoensis* exhibited a distinct electrophoretic profile.

Under reducing conditions, the banding patterns showed a high degree of similarity among all species in the molecular weight distribution of the protein bands. Prominent bands were observed at approximately 14.2, 20, 26, and 45 kDa, differing mainly in their staining intensity (Figure 3D). These bands can suggest snake venom serine proteinases (SVSPs), typically in the 25–35 kDa range, with *B. jararacussu* showing reduced intensity compared to *B. atrox*. Despite the overall similarity among the venoms, a greater resemblance was observed among *B. alternatus*, *B. atrox*, and *B. jararaca*, as well as between *B. marajoensis* and *B. neuwiedi*. These findings corroborate the idea that interspecific variation in the abundance of venom compounds underlies the functional results presented.

### 2.2. Antivenom Recognition

All snake venoms were evaluated for immunorecognition by bothropic antivenom, in order to determine whether the pentavalent antivenom exhibited cross-reactivity toward the tested venoms. All venoms showed significant immunoreactivity against the antivenom, demonstrating broad antigenic recognition (Figure 4). In contrast, non-immune horse serum exhibited no significant binding to the venoms, indicating the absence of nonspecific interactions and confirming the specificity of the observed antibody-mediated recognition.

Remarkably, *B. marajoensis* exhibited significantly higher recognition than all other *Bothrops* spp. venoms tested, suggesting a greater degree of antigenic similarity and/or a higher abundance of shared immunoreactive components among the venoms evaluated.

### 2.3. Phosphodiesterase Activity

Snake venom phosphodiesterases (PDEs) play important synergistic roles in envenomation by modulating vascular responses and purinergic signaling [12,13]. To investigate this less explored enzymatic component, relative PDE activity was evaluated by the specific substrate use among the analyzed venoms (Figure 5). Notably, *B. marajoensis* venom exhibited higher PDE activity compared to *B. atrox*, *B. jararaca* and *B. alternatus* venoms. Regarding the other venoms, *B. neuwiedi*, *B. moojeni*, and *B. jararacussu*, *B. marajoensis* venom showed significantly lower PDE activity. These results indicate differences in venom composition and reveal interspecific biochemical variability among the analyzed venoms.

### 2.4. Effects of Bothrops Venoms on the Viability of Differentiated C2C12 Myotubes

Although isolated myotoxins have been identified in *B. marajoensis* venom, the overall myotoxic effect of the crude venom remains poorly understood. To address this, we evaluated the direct effects of *Bothrops* venoms on differentiated C2C12 myotubes.

The evaluated *Bothrops* venoms exhibited distinct effects on the viability of differentiated C2C12 myotubes (Figure 6). The strongest reductions were observed for *B. jararacussu* and *B. moojeni*, while *B. atrox* also produced a marked effect, although with considerable inter-replicate variability. Conversely, *B. alternatus* and *B. marajoensis* maintained comparatively higher viability values, and *B. jararaca* exhibited an intermediate response. Owing to the limited number of concentrations tested, the effect of *B. neuwiedi* could not be fully defined.

Cell viability was differentially affected by the venom species and the amount applied per well. At the highest concentration, *B. jararacussu* venom induced the most pronounced reduction in cell viability, reaching 3.107 ± 2.187%, followed by *B. atrox* and *B. moojeni*, with viability values of 27.080 ± 29.472% and 40.530 ± 4.271%, respectively. In contrast, cells exposed to *B. marajoensis* venom maintained the highest viability at this concentration, at 84.603 ± 2.399%.

A progressive recovery of cell viability with decreasing venom amounts was particularly evident for *B. jararacussu*, increasing from 3.107 ± 2.187% at 50 µg/mL to 70.390 ± 9.212% at 6.25 µg/mL. A similar concentration-related pattern was observed for *B. jararaca*, for which viability increased from 56.090 ± 3.946% to 95.433 ± 4.425%, and for *B. alternatus*, which increased from 65.650 ± 10.698% to 102.200 ± 2.971%. The latter value was comparable to that of the negative control, indicating no apparent reduction in cell viability at the lowest tested amount.

For *B. atrox*, marked reductions in viability were observed at 50 and 25 µg/mL (10 and 5 µg/well); however, these conditions exhibited high standard deviations, indicating substantial variability among replicates. At lower venom amounts, viability increased to 78.173 ± 4.423% and 88.833 ± 5.277% at 12.5 and 6.25 µg/mL (2.5 and 1.25 µg/well), respectively. *B. moojeni* caused a persistent reduction in viability across the tested range, although partial recovery was observed at 6.25 µg/mL. Conversely, *B. marajoensis* maintained viability values between approximately 74% and 85%, without a clear concentration-dependent recovery. *B. neuwiedi*, evaluated only at 12.5 and 6.25 µg/mL (2.5 and 1.25 µg/well), produced viability values of 51.540 ± 21.846% and 66.700 ± 44.100%, respectively, with considerable experimental variability.

The venoms exhibited distinct concentration–response profiles, with the most pronounced reductions in ATP-dependent cell viability observed following exposure to *B. jararacussu*, *B. moojeni*, *B. atrox*, and *B. jararaca*. In contrast, cell viability remained above 50% throughout the evaluated concentration range for *B. alternatus* and *B. marajoensis*. For *B. neuwiedi*, only the two lowest concentrations were evaluated due to the limited amount of venom available, which prevented testing the complete concentration range used for the other venoms. Consequently, a reliable determination of the half-maximal inhibitory concentration (IC_50_) was not possible for this venom. For the remaining venoms, IC_50_ values were estimated from the concentration–response data and expressed in µg/mL, based on a final assay volume of 200 µL per well. The estimated IC_50_ values are presented in Table 1.

Overall, the findings indicate that the effects of *Bothrops* venoms on differentiated C2C12 myotubes were species-dependent and, for some venoms, dose-dependent. Under the present experimental conditions, *B. jararacussu* and *B. moojeni* showed the strongest apparent effects on ATP-dependent viability and the lowest estimated IC_50_ values. These results are compatible with comparatively greater apparent cytotoxic or metabolically inhibitory activity toward differentiated skeletal muscle cells. Although *B. atrox* also produced substantial reductions in ATP-dependent viability, the high variability and non-monotonic response observed at the two highest concentrations prevent a definitive interpretation of its relative potency. Therefore, its estimated IC_50_ should be regarded as preliminary and interpreted with particular caution.

In contrast, *B. jararaca*, *B. alternatus*, and *B. marajoensis* did not reduce viability below 50% within the evaluated concentration range. This finding suggests lower apparent potency in this experimental model compared with *B. jararacussu* and *B. moojeni*. However, it does not demonstrate the absence of biological effects, as changes in cellular function may occur even when the ATP-dependent signal remains above 50%.

The results obtained for *B. neuwiedi* remain inconclusive because only two concentrations were tested and the responses exhibited substantial variability. Additional concentrations, particularly above 12.5 µg/mL, would be required to establish whether this venom induces a concentration-dependent reduction in C2C12 myotube viability.

It should also be emphasized that the ATP-based assay primarily reflects cellular metabolic activity. Consequently, a reduction in luminescence may represent a decrease in the number of viable cells, suppression of cellular metabolism, or a combination of both processes. The results should therefore not be interpreted exclusively as direct evidence of cell death. Complementary analyses of membrane integrity, cellular morphology, mitochondrial activity, and cell-death pathways would be necessary to better characterize the mechanisms underlying the observed effects.

The present findings provide preliminary evidence of marked interspecific differences in the effects of *Bothrops* venoms on differentiated C2C12 myotubes. *B. jararacussu* and *B. moojeni* produced the most consistent reductions in ATP-dependent viability and displayed the lowest estimated IC_50_ values among the venoms evaluated. *B. atrox* also showed a potentially relevant effect, although its concentration–response profile was affected by substantial experimental variability. In comparison, *B. jararaca*, *B. alternatus*, and *B. marajoensis* exhibited lower apparent potency within the tested concentration range, whereas the available data for *B. neuwiedi* were insufficient for a reliable assessment.

## 3. Discussion

Although the venoms of *Bothrops* species share a common repertoire of toxin families, including SVMPs, PLA_2_s, SVSPs, LAAOs, C-type lectins (CTLs), and cysteine-rich secretory proteins (CRISPs), substantial interspecific variation exists in the relative abundance of these components. Such compositional differences are considered of *Bothrops* venoms and have been associated with ecological adaptation, prey specialization, geographic distribution, and evolutionary divergence [14,15]. From a phylogenetic perspective, *B. marajoensis* belongs to the *B. atrox* species complex, sharing a close evolutionary lineage with *B. atrox*, *B. moojeni*, and *B. jararacussu*, whereas *B. neuwiedi* and *B. jararaca* represent the *B. neuwiedi* + *B. jararaca* clade, and *B. alternatus* represent distinct clade [16]. This evolutionary proximity is reflected in the overall chromatographic and electrophoretic similarities among members of the *B. atrox* complex observed in this study. However, the distinct quantitative variations and functional differences, such as the lower apparent myotoxicity of *B. marajoensis* compared to *B. moojeni*, highlight that venom diversification within *Bothrops* snakes is shaped not only by phylogenetic relationships but also by local evolutionary pressures, geographic isolation, as *B. marajoensis* in Marajó Island (Brazil), and quantitative regulation of toxin expression.

In the present study, the chromatographic profiles and SDS-PAGE revealed a high degree of similarity among the analyzed venoms, indicating that these species possess largely conserved venom arsenals. Nevertheless, distinct differences in peak intensities were observed, particularly in the central region of the chromatograms. Notably, *B. jararacussu* exhibited a marked increase in the intensity of components eluting in the intermediate portion of the profile, suggesting a higher abundance of specific toxin classes rather than the presence of exclusive proteins. This observation is consistent with previous proteomic studies demonstrating that venom diversification within the genus is driven primarily by quantitative shifts in toxin expression rather than by the emergence or disappearance of toxin families [14,15].

*B. jararacussu* represents an interesting example of quantitative venom specialization. Previous studies demonstrated that adult specimens express substantially higher levels of Lys49 PLA_2_ myotoxins, whereas juvenile venoms contain proportionally higher levels of proteolytic enzymes. This ontogenetic shift highlights the remarkable plasticity of venom composition within a single species and reinforces that differences in chromatographic peak intensity can reflect biologically meaningful changes in toxin abundance. The higher intensity observed in the intermediate chromatographic region of *B. jararacussu* in the present study is consistent with its well-known enrichment in PLA_2_ myotoxins, which contribute significantly to the pronounced myotoxic activity characteristic of this species [15].

The present study demonstrated that *B. marajoensis* venom shares the conserved biochemical architecture characteristic of the genus while exhibiting differences in protein composition and enzymatic activity. These findings are consistent with previous venomic studies showing that *Bothrops* venoms are predominantly composed of conserved toxin families, including SVMPs, PLA_2_s, and SVSPs, whose relative abundance varies among species and contributes to functional diversification rather than the emergence of novel toxin classes [17,18,19].

A major finding of the present study was the broad immunoreactivity of the commercial bothropic antivenom against all analyzed *Bothrops venoms*. Remarkably, *B. marajoensis* venom exhibited the highest recognition by the commercial antivenom, despite not being included in the pentavalent immunization mixture (which consists of *B. alternatus*, *B. jararaca*, *B. jararacussu*, *B. moojeni* and *B. neuwiedi*) [20]. Despite the biochemical differences revealed by chromatographic and electrophoretic analyses, ELISA demonstrated extensive antigenic recognition, indicating that the major immunogenic epitopes are highly conserved among these species, suggesting also that *B. marajoensis* shares highly conserved immunodominant epitopes with the species used for antivenom production. Furthermore, quantitative variations in venom composition, such as a higher relative proportion of major immunogens like SVMPs and PLA_2_s, may result in greater overall antibody binding capacity per microplate well during indirect ELISA. This observation agrees with previous antivenomic studies showing that SVMPs, SVSPs, and PLA_2_s are the principal immunogens during horse immunization and are largely responsible for the extensive cross-reactivity exhibited by Brazilian bothropic antivenoms [18,19]. The predominance of these conserved toxin families in *B. marajoensis* venom likely explains its efficient recognition by the commercial antivenom, despite quantitative variation among species [18]. Although ELISA does not directly predict toxin neutralization, broad antigen recognition represents an essential prerequisite for antivenom efficacy. Therefore, complementary antivenomic approaches combined with in vivo neutralization assays would further clarify the effectiveness of the commercial bothropic antivenom against *B. marajoensis* venom, correlating with functional protection against lethality and local tissue damage.

Another relevant finding was the marked interspecific variation in PDE activity. *B. marajoensis* exhibited intermediate PDE activity compared with the other *Bothrops* species analyzed, indicating that enzymatic activity does not necessarily correlate with the overall chromatographic or electrophoretic similarity among venoms. Although PDEs are minor venom components, they have been associated with nucleotide hydrolysis, adenosine generation, platelet dysfunction, vascular alterations, and modulation of inflammatory responses, acting synergistically with other toxin families during envenoming [12,13]. These findings support the concept that venom diversification involves not only variation in major toxin families but also modulation of accessory enzymes that contribute to the functional diversity of *Bothrops* venoms.

One of the most relevant functional findings of the present study was the marked interspecific variability in the effects of *Bothrops* venoms on differentiated C2C12 myotubes. Skeletal muscle represents one of the primary targets of local tissue injury following *Bothrops* envenoming, and differentiated C2C12 myotubes constitute a well-established in vitro model for investigating the direct effects of venom toxins on skeletal muscle because they reproduce several structural and metabolic characteristics of mature muscle fibers [21]. Under the experimental conditions employed, *B. jararacussu* and *B. moojeni* induced the greatest reductions in ATP-dependent viability, whereas *B. marajoensis* exhibited a lower impact on myotube viability, even at the highest venom concentration tested. These findings demonstrate that, although *Bothrops* venoms share the same major toxin families, they differ considerably in their biological activities, emphasizing that quantitative and qualitative variations in venom composition are critical determinants of toxicity [22].

The significant cytotoxicity seen in *B. jararacussu* aligns with its known high levels of myotoxic phospholipases A_2_, especially Lys49 PLA_2_ homologues, recognized as some of the most potent myotoxins in *Bothrops* venoms [23]. These Lys49 homologues, unlike catalytically active Asp49 PLA_2_s, cause toxicity via a mechanism that does not rely on catalysis. They disrupt plasma membrane integrity, leading to swift calcium influx, mitochondrial failure, ATP depletion, oxidative stress, and ultimately irreversible myonecrosis [21,23]. Likewise, the marked cytotoxicity induced by *B. moojeni* probably reflects the combined action of abundant PLA_2_ myotoxins together with metalloproteinases, which contribute to extracellular matrix degradation and potentiate muscle damage by facilitating toxin diffusion and amplifying local tissue injury [24].

In contrast, *B. marajoensis* exhibited comparatively low cytotoxicity, maintaining approximately 75–85% cell viability throughout the concentration range evaluated. Although previous studies have identified myotoxic Lys49 PLA_2_ homologues such as BmjeTX-I, BmjeTX-II and BmajPLA_2_-II in this venom [5]. The present findings suggest that differences in the relative abundance of these toxins, their intrinsic toxic potency, or synergistic interactions with other venom components may contribute to the reduced myotoxic profile observed in comparison with *B. jararacussu* and *B. moojeni.* Interestingly, this functional behavior is compatible with the RP-FPLC chromatographic profiles obtained in the present study, which revealed distinct distributions of venom components among the analyzed species. Together, these observations reinforce the concept that biological activity depends not only on the presence of particular toxin families but also on their relative abundance and interactions within the venom proteome [22].

It is important to note that the CellTiter-Glo assay detects intracellular ATP, indicating cellular metabolic activity rather than immediate cell death. Therefore, reduced luminescence could be due to mitochondrial issues, membrane damage, metabolic suppression, or activated cell death pathways. Conversely, maintained ATP levels do not always mean the cell is unharmed, as sublethal injury might still be present [25]. The cell viability assays demonstrate that the myotoxic potential of *Bothrops* venoms varies substantially among species despite the conservation of their principal toxin families. The comparatively low cytotoxicity exhibited by *B. marajoensis* highlights that relatively subtle differences in venom composition are sufficient to generate distinct biological responses. These findings complement the biochemical characterization presented herein and reinforce the importance of integrating proteomic and functional approaches to improve our understanding of venom variability and its pathological consequences, particularly for medically relevant but comparatively understudied species such as *B. marajoensis*.

Overall, this study expands the biochemical characterization of the poorly investigated *B. marajoensis* venom by integrating chromatographic, electrophoretic, immunological, and enzymatic analyses. Despite quantitative differences in toxin abundance and PDE activity, the conservation of major toxin families preserved broad antigenic recognition by the commercial bothropic antivenom, supporting its potential clinical applicability. Future LC-MS/MS-based proteomic studies associated with functional neutralization assays will be important to identify toxin isoforms responsible for the observed biochemical differences and to improve our understanding of venom evolution, antivenom efficacy, and the biotechnological potential of *B. marajoensis* venom.

## 4. Materials and Methods

### 4.1. Venoms

Venom from seven adults *Bothrops* species from captive bred, *B. alternatus*, *B. atrox*, *B. jararaca*, *B. jararacussu*, *B. marajoensis*, *B. moojeni*, and *B. neuwiedi*, were obtained from the Serpentarium of the University of São Paulo at Ribeirão Preto Medical School, which is accredited by the Instituto Brasileiro do Meio Ambiente e dos Recursos Naturais Renováveis (Brazilian Institute of Environment and Renewable Natural Resources), under the registration number 1506748. This research project is registered at the Sistema Nacional de Gestão do Patrimônio Genético e do Conhecimento Tradicional Associado (SISGEN, National System of Genetic Heritage and Associated Traditional Knowledge, No. A4A9FDD). Venoms were dried under vacuum at room temperature for 6 h and stored at −20 °C until their use.

### 4.2. Venom Fractionation

Dried venom (3 mg) was dissolved in 2 mL of deionized water and centrifuged at 13,000 rpm for 10 min, at 4 °C. The supernatant was then collected, and trifluoroacetic acid (TFA) and acetonitrile (MeCN) were added to achieve final concentrations of 0.1% (*v*/*v*) and 5% (*v*/*v*), respectively. Purification was performed by reversed-phase protein liquid chromatography (RP-HPLC), using a C18 analytical column (250 × 4.6 mm, 5 μm, Phenomenex, Torrance, CA, USA), in an ÄKTA Purifier UPC10 system (GE Healthcare, Uppsala, Sweden). The samples were fractionated according to the method described by Calvete et al. (2007) [17]. The column was pre-equilibrated with solution A (0.1% TFA), and the proteins were eluted with a segmented gradient up to 100% solvent B (0.1% TFA in 80% ACN), at a flow rate of 1.0 mL/min. Fraction elution was monitored at 214 nm.

### 4.3. Electrophoresis

*Bothrops* spp. species venom (50 μg) was subjected to 13.5% SDS-PAGE and to 10% Tricine-SDS-PAGE, according to the method described by Laemmli (1970) [26] and Schägger (2006) [27], respectively, both under reducing and non-reducing conditions. As markers, Full Range Molecular Weight (250–10 kDa, 161-0374, BioRad, Hercules, CA, USA), Low Molecular Weight (97.0–14.4 kDa, 17-0446-01, GE Healthcare, Uppsala, Sweden) and Ultra-Low Molecular Weight (26.6–1.06 kDa, M3546-1VL, Sigma-Aldrich, Saint Louis, MO, USA) were used. All gels were stained with Coomassie^®^ Brilliant Blue G-250 (Sigma-Aldrich, St. Louis, MO, USA).

### 4.4. ELISA

Indirect enzyme-linked immunosorbent assay (ELISA) was performed according to Oliveira et al. (2018) [28]. 96-well microplate (Costar, Corning Incorporated, Corning, NY, USA) was coated with *Bothrops* spp. venoms (2 μg each) in 0.05 M carbonate/bicarbonate buffer, pH 9.6 (100 μL/well). Non-immunized horse serum was coated and used as positive control (1:50, H0146, lot SLBS7574, Sigma-Aldrich, St. Louis, MO, USA). The plate was incubated for 16 h at 4 °C, then washed three times with phosphate-buffered saline (PBS), pH 7.2, and blocked by adding 250 μL of PBS containing 2% (*w*/*v*) non-fat dry milk (ref. 1706404, BioRad, Lagoa Santa, Brazil; MPBS) and incubated for 2 h at 37 °C. The plate was then washed three times with PBS-0.05% Tween (PBS-T) and three times with PBS and incubated for 1 h at 37 °C with commercial bothropic antivenom (1:100 in 1% MPBS, batch 1305077, Instituto Butantan, São Paulo, Brazil). As a negative control, venoms were incubated with non-immunized horse serum diluted 1:100 in 1% MPBS. The plate was washed three times with PBS-T and PBS, respectively. After that, the plate was incubated with 100 μL of anti-horse polyclonal antibodies conjugated with peroxidase (IgG-HRP, A6917, Sigma-Aldrich, St. Louis, MO, USA) diluted 1:10,000 in 1% MPBS. After 1 h of incubation at 37 °C, the plate was washed three times with PBS-T and three times with PBS. To each well, 100 μL of 3,3′,5,5′-Tetramethylbenzidine (TMB, ref. T0440, Sigma-Aldrich, St. Louis, MO, USA), and incubated in a dark chamber at RT until color development. The reaction was stopped with 50 μL of 1 M H_2_SO_4_ (Merck, São Paulo, Brazil). Absorbance was measured at 405 nm on a 96-well plate reader (Sunrise-basic Tecan, Männedorf, Switzerland). The assay was performed in quadruplicates (n = 4).

### 4.5. PDE Activity

Phosphodiesterase (PDE) activity was determined in *Bothrops* spp. venoms according to Björk (1963) [29], modified by Valério et al. (2002) [30], in a 96-well microplate. *Bothrops* spp. venoms (5 μg/well) were incubated in 0.1 M Tris–HCl buffer, pH 8.0, 10 min at 37 °C. A solution containing 1 mM *bis*(*p*-nitrophenyl) phosphate (N3002, Sigma-Aldrich, St. Louis, MO, USA) substrate was added, and the plate was incubated for 30 min at 37 °C. The reaction was stopped with 0.05 M NaOH, and absorbance readings were performed in a microplate reader (Sunrise-basic, Tecan, Männedorf, Switzerland) at 400 nm. The highest absorbance was considered 100% of activity. The assay was carried out in quadruplicate (n = 4).

### 4.6. C2C12 Cell Culture and Myogenic Differentiation

Murine C2C12 myoblasts (ATCC CRL-1772), kindly provided by A.M.S. from Instituto Butantan (São Paulo, Brazil), were received cryopreserved and subsequently thawed and expanded under aseptic conditions. The cells used in the experiments were cultured for one passage after thawing. Since the cells were provided by another laboratory, their complete previous passage history was not available.

Cells were maintained in cell culture flasks in modified RPMI 1640 medium (Nova Biotecnologia Ltd.a., São Paulo, Brazil; BR30260-05) containing L-glutamine (2.05 mM), HEPES (25 mM), β-mercaptoethanol (50 µM), sodium bicarbonate (2.0 g/L), and 1% (*v*/*v*) penicillin/streptomycin, corresponding to final concentrations of approximately 100 U/mL penicillin and 100 µg/mL streptomycin. The medium was supplemented with 10% (*v*/*v*) fetal bovine serum (FBS; Nova Biotecnologia Ltd.a., Brazil), which was heat-inactivated in the laboratory before use. Cells were maintained at 37 °C in a humidified atmosphere containing 5% CO_2_ in a Panasonic MCO-19AIC-PA CO_2_ incubator (Panasonic, Mississauga, ON, Canada). Cell morphology and growth were routinely monitored by phase-contrast microscopy.

When cultures reached approximately 80% confluence, the culture medium was removed and the adherent cell monolayer was exposed to 5 mL of 0.25% trypsin-EDTA, 1× (Nova Biotecnologia Ltd.a., Brazil), for approximately 5 min or until complete cell detachment was observed. Trypsin activity was then neutralized by adding 10 mL of modified RPMI 1640 medium supplemented with 10% FBS. The resulting cell suspension was transferred to a centrifuge tube and centrifuged for 10 min at 2500 rpm using an Eppendorf Centrifuge 5810 R equipped with an A-4-44 rotor (Eppendorf, Hamburg, Germany). The supernatant was discarded, and the resulting cell pellet was resuspended in 1 mL of complete culture medium.

Cell concentration and viability were determined using a Neubauer counting chamber after staining with 0.4% Trypan Blue solution (Bio-Rad Laboratories, Hercules, CA, USA). Cells were then seeded into cell culture-treated 96-well plates at a density of 5 × 10^4^ cells/well and maintained under the same incubation conditions. Approximately three days after seeding, the cultures reached ~80% confluence.

Myogenic differentiation was initiated by replacing the growth medium with differentiation medium consisting of the same modified RPMI 1640 formulation containing L-glutamine (2.05 mM), HEPES (25 mM), β-mercaptoethanol (50 µM), sodium bicarbonate (2.0 g/L), and penicillin/streptomycin, but without FBS and supplemented instead with 2% (*v*/*v*) horse serum (Nova Biotecnologia Ltd.a., Brazil). Horse serum was also heat-inactivated in the laboratory before use. Cells were maintained under differentiation conditions for 72 h, during which progressive cell alignment, myoblast fusion, and the formation of elongated multinucleated myotubes were observed, confirming the establishment of a differentiated skeletal muscle cell model.

#### 4.6.1. Treatment with *Bothrops* Venoms

After the differentiation period, C2C12-derived myotubes were exposed to crude venoms from all seven *Bothrops* species. Before treatment, the culture medium was completely removed from all wells, including the negative control wells, and replaced with fresh medium. Venom stock solutions were individually diluted immediately before use in the same modified RPMI 1640 medium used during differentiation, supplemented with 2% (*v*/*v*) horse serum. For six of the venoms, final concentrations of 6.25, 12.5, 25, and 50 µg/mL were tested in a final volume of 200 µL per well. For *B. neuwiedi*, only the two lowest concentrations (6.25 and 12.5 µg/mL) were evaluated due to the limited amount of venom available. The assay was performed in triplicate (n = 3). Negative control myotubes underwent the same complete medium replacement procedure and received 200 µL of fresh venom-free culture medium. Differentiated myotubes were exposed to the respective venom treatments for 24 h at 37 °C in a humidified atmosphere containing 5% CO_2_. At the end of the exposure period, cell viability was assessed using an ATP-based luminescence assay.

#### 4.6.2. Cell Viability Assay

Cell viability was determined using the CellTiter-Glo^®^ 2.0 Luminescent Cell Viability Assay (Promega, Madison, WI, USA), following the manufacturer’s recommendations. The assay is based on the quantification of intracellular adenosine triphosphate (ATP), which serves as an indicator of metabolically active and viable cells.

During the 24 h venom exposure period, cells were maintained in 200 µL of modified RPMI 1640 medium supplemented with 2% (*v*/*v*) horse serum. At the end of the exposure period, 150 µL of culture medium was carefully removed from each well, leaving 50 µL of medium containing the respective venom treatment. Subsequently, 50 µL of CellTiter-Glo^®^ 2.0 reagent was added directly to each well of the original cell culture plate, resulting in a 1:1 ratio of assay reagent to the remaining culture medium and a final assay volume of 100 µL per well.

After reagent addition, the original culture plate was gently agitated manually by lightly tapping the sides of the plate to promote cell lysis, ATP release, and homogenization of the reaction mixture. The entire 100 µL content of each well was then transferred to a white 96-well plate (Thermo Scientific, Waltham, MA, USA) for luminescence measurement. Following signal stabilization, luminescence was measured using a GloMax^®^ Explorer Multimode Microplate Reader (Promega, Madison, WI, USA). Signal acquisition was performed using the pre-programmed CellTiter-Glo/Luminescence protocol available on the instrument and corresponding to the CellTiter-Glo^®^ assay. Cell viability was expressed as a percentage relative to the negative control, which was considered to represent 100% viability.

### 4.7. Statistical Analyses

Quantitative results are expressed as mean ± standard deviation (SD) values. For statistical comparisons involving three or more groups, one-way analysis of variance (ANOVA) was performed, followed by Tukey’s post hoc test for multiple pairwise comparisons or Dunnett’s test when comparing treatments against a control group. A value of *p* < 0.05 was considered statistically significant. All analyses were conducted using GraphPad Prism software (version 10.0, GraphPad Software, San Diego, CA, USA).

## 5. Conclusions

This study provides an expanded characterization of *B. marajoensis* venom through comparative analyses with six venoms from *Bothrops* species. Although they shared a conserved biochemical composition, chromatographic and electrophoretic analyses revealed quantitative differences in protein venoms, reflecting distinct enzymatic and biological profiles. Despite these biochemical differences, the antivenom recognized all venoms, supporting the conservation of major antigenic determinants across the genus. Together, these findings contribute to the understanding of venom variability in *B.* genus, reinforce the potential clinical applicability of the bothropic antivenom against *B. marajoensis*, and provide a framework for future proteomic studies, toxin isolation, and functional investigations aimed at identifying the molecular determinants responsible for the distinct biological activities of this poorly explored venom.

## Figures and Tables

**Figure 1:**
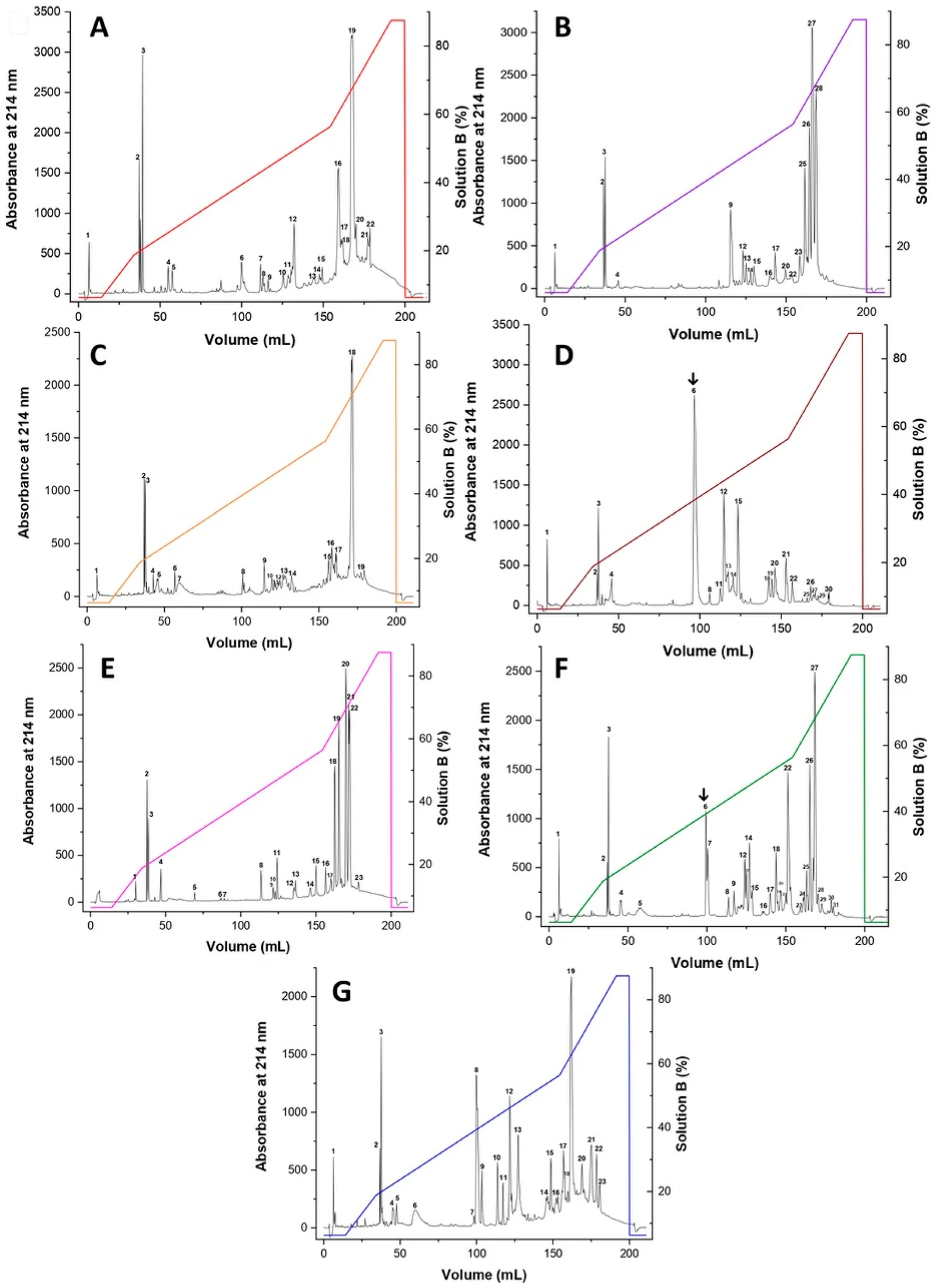
Reversed-phase chromatographic profiles of *Bothrops* spp. venoms. Crude venoms from *Bothrops* species were fractionated by reversed-phase liquid chromatography. Profiles correspond to: (**A**) *B. alternatus* (red), (**B**) *B. atrox* (purple), (**C**) *B. jararaca* (orange), (**D**) *B. jararacussu* (brown), (**E**) *B. marajoensis* (pink), (**F**) *B. moojeni* (green), and (**G**) *B. neuwiedi* (blue). Elution was monitored at 214 nm, using a C18 analytical column, and a segmented gradient of up to 100% solvent B (0.1% TFA in 80% MeCN), at a flow rate of 1.0 mL/min. The arrows indicate the intermediate peak 6.

**Figure 2:**
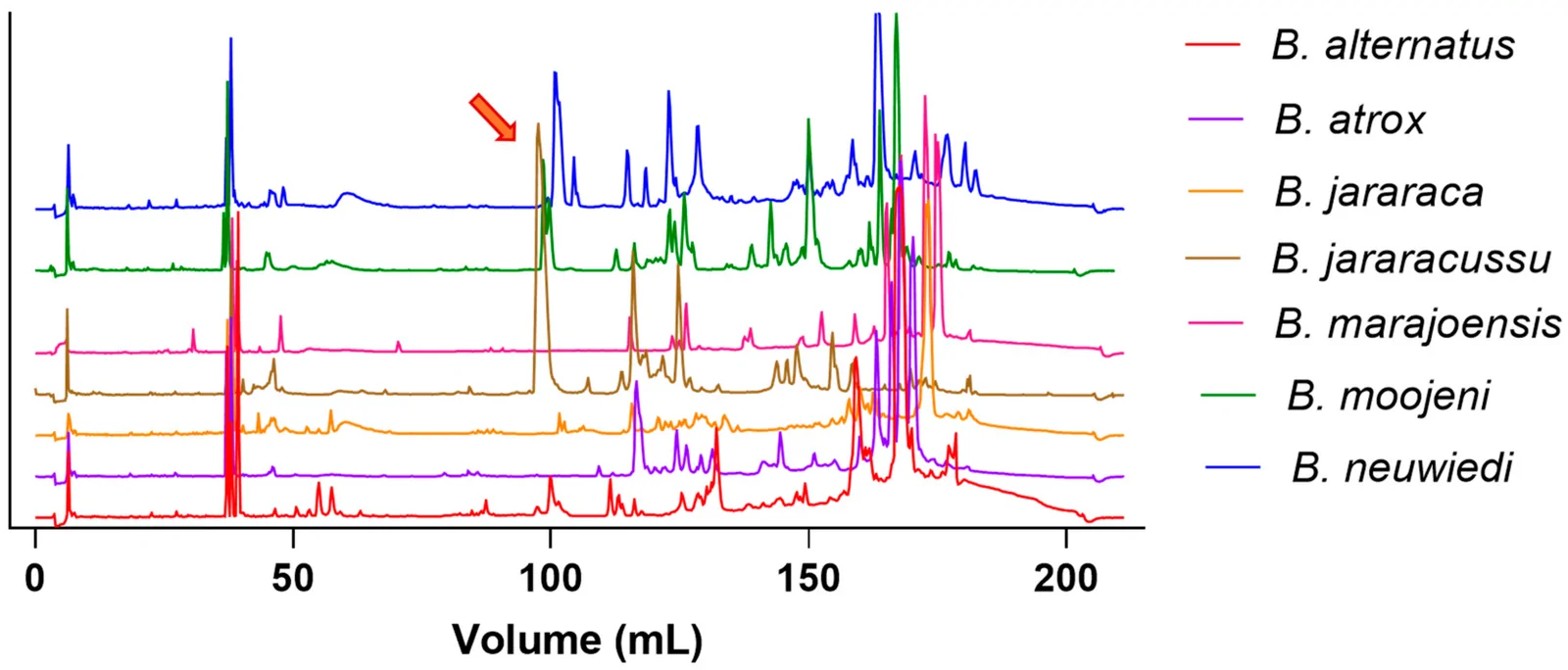
Comparative chromatographic profiles of crude venoms from seven *Bothrops* species obtained by reversed-phase chromatography. Chromatograms were normalized and vertically offset for clarity. Distinct elution profiles reflect differences in the molecular weight distribution and relative abundance of venom components among species. Elution volume (mL) is shown on the x-axis. The arrow indicates the predominant intermediate-eluting peak in this region.

**Figure 3:**
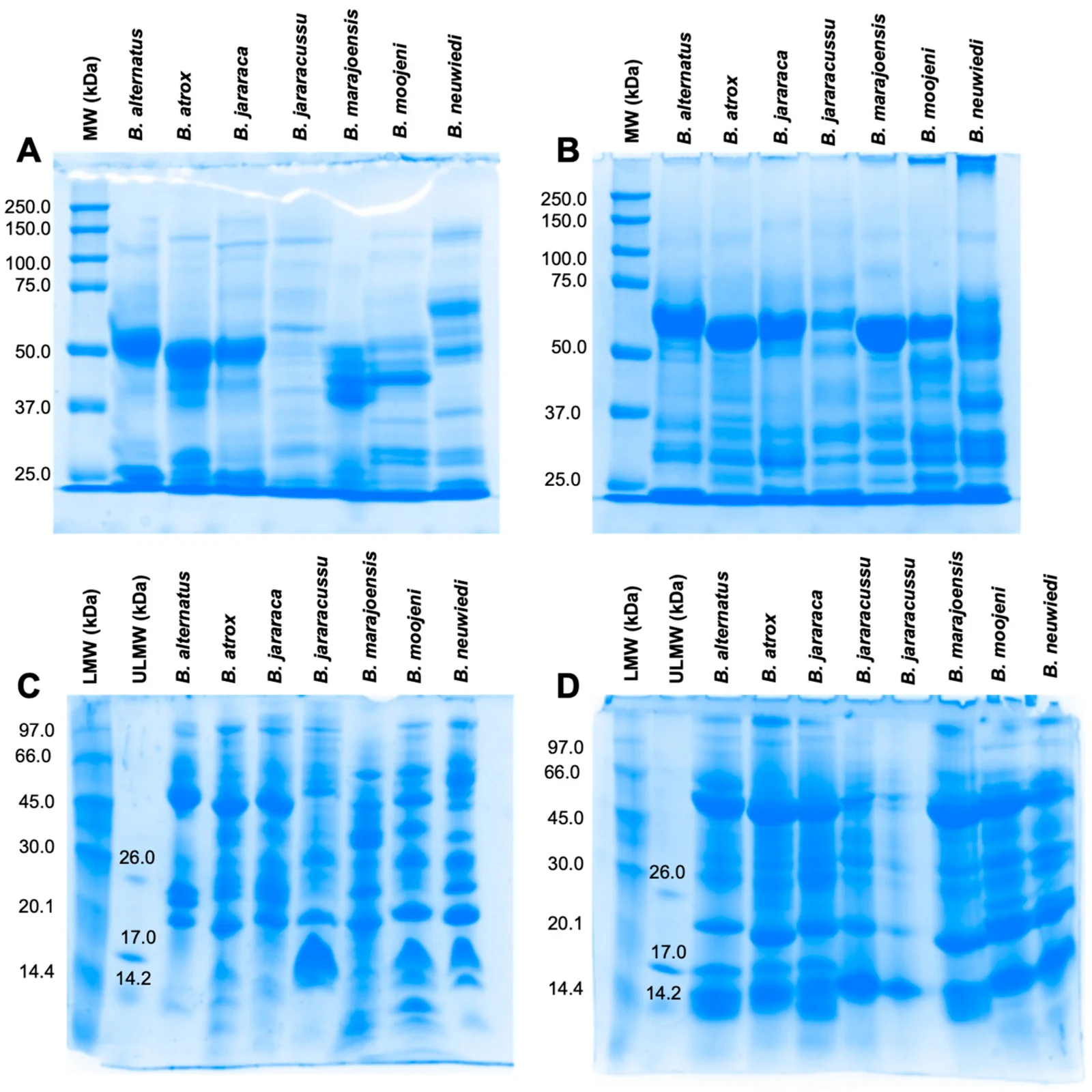
Electrophoretic profiles of *Bothrops* spp. venoms. Venoms (50 μg) were analyzed by (**A**) Non-reduced 13.5% SDS-PAGE; (**B**) reduced 13.5% SDS-PAGE; (**C**) non-reduced 10% Tricine SDS-PAGE; (**D**) reduced 10% Tricine SDS-PAGE. MW: molecular weight marker (250–10 kDa); LMW: low-molecular-weight marker (97.0–14.4 kDa); ULMW: ultra-low-molecular-weight marker (26.6–1.06 kDa).

**Figure 4:**
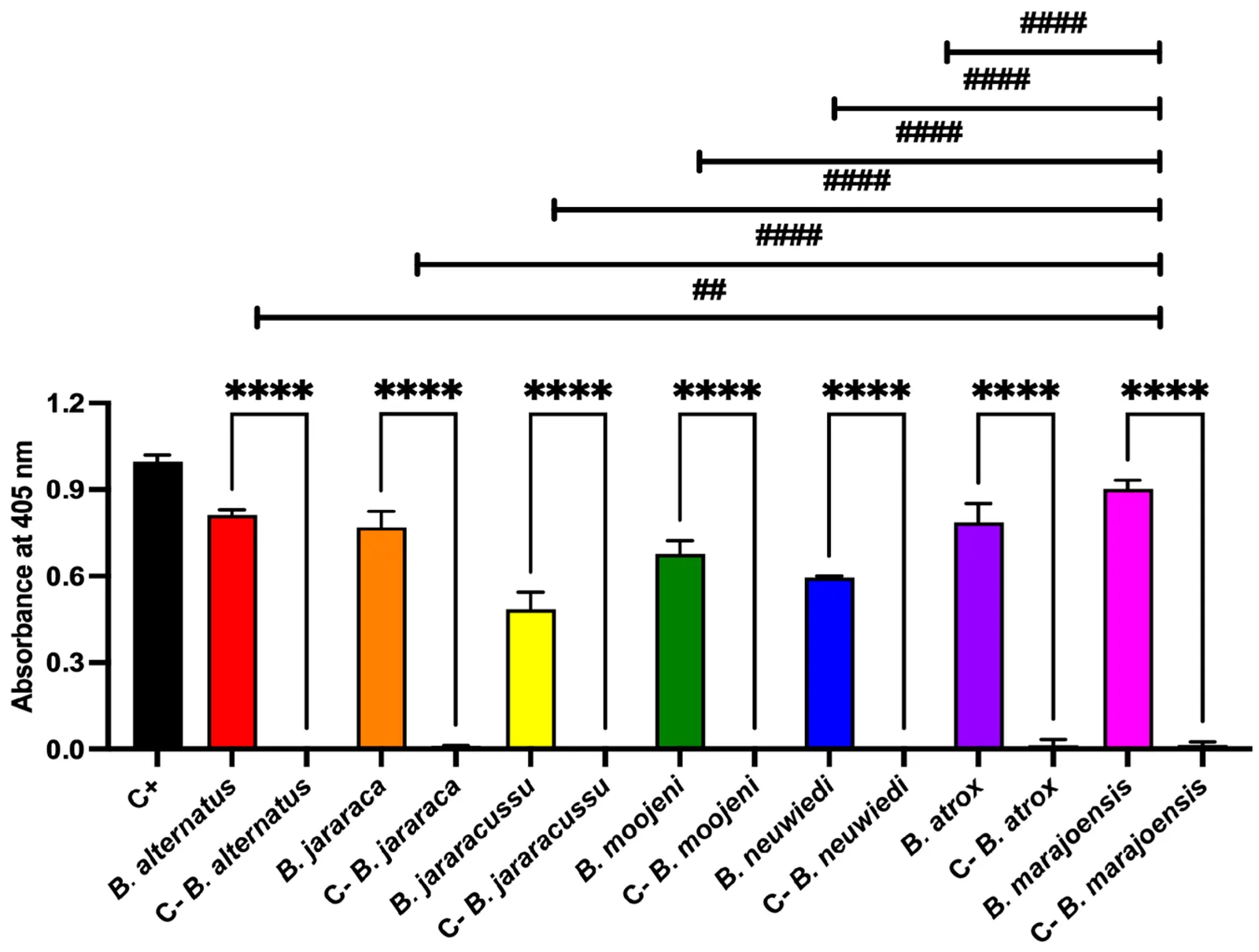
*Bothrops* spp. venoms recognition by bothropic antivenom. Through indirect ELISA, *Bothrops* spp. venom (2 μg each) were incubated with commercial bothropic antivenom from Instituto Butantan. Absorbance was measured at 405 nm. Data are presented as mean ± SD analyzed by one-way ANOVA and Tukey’s multiple comparison test (n = 4). C+: wells coated with non-immunized horse serum diluted 1:50; C− venom: venoms incubated with non-immunized horse serum diluted 1:100. **** *p* < 0.0001 when compared to its negative control. ## *p* < 0.003 and #### *p* < 0.0001 when compared to *B. marajoensis*.

**Figure 5:**
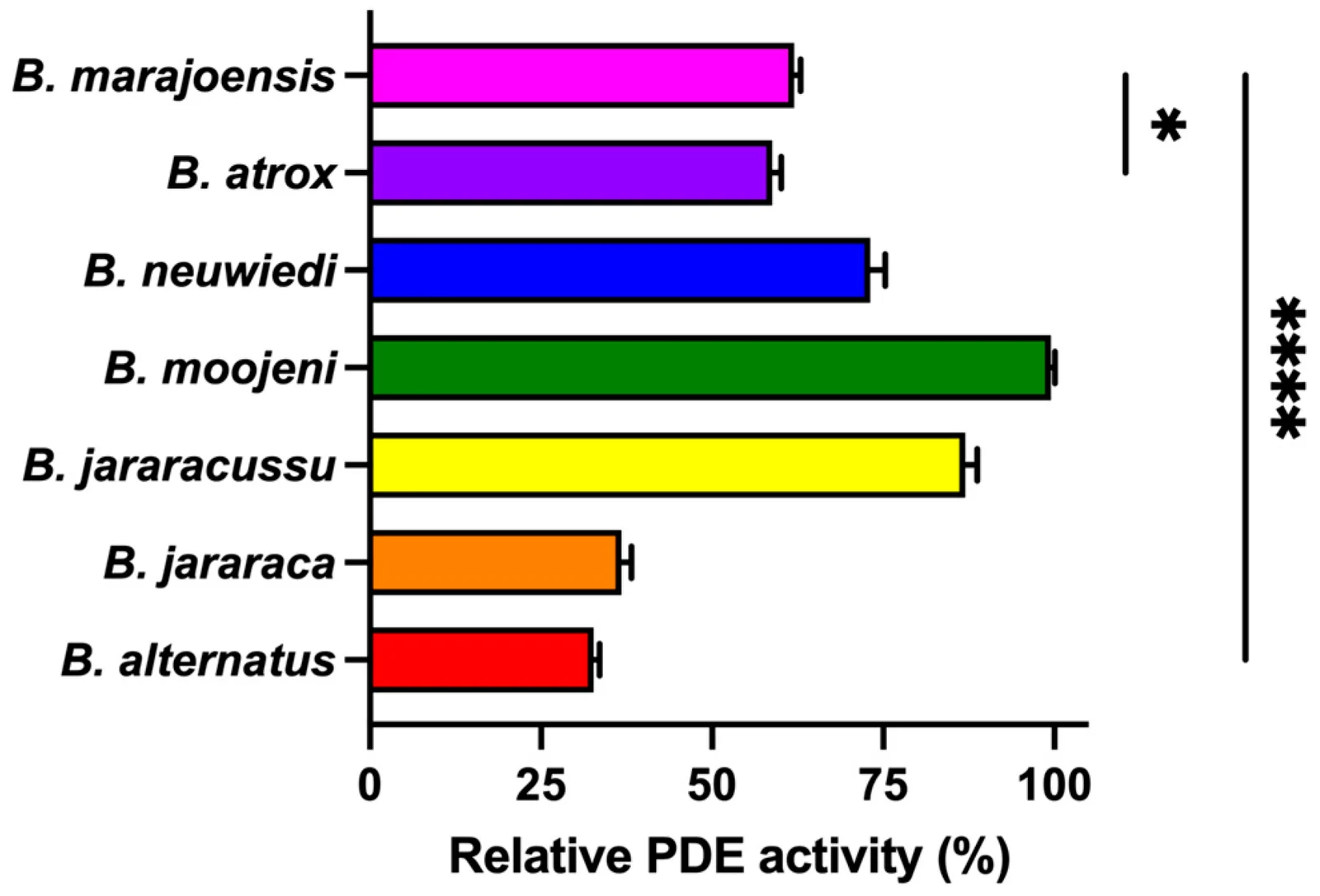
PDE activity assay with *Bothrops* spp. venoms. A mixture (150 μL) of venoms (5 μg) and 0.1 M Tris-HCl, pH 8, was incubated for 10 min at 37 °C. The substrate *bis*(*p*-nitrophenyl) phosphate (1 mM) was added, and was incubated for 30 min at 37 °C. The reaction was stopped with 0.05 M sodium hydroxide (NaOH). Absorbance was measured at 400 nm. The enzymatic activity was expressed as a percentage of relative activity to that of highest absorbance. Bars represent the mean ± SD (n = 4). Data were analyzed by one-way ANOVA followed by Dunnett’s post hoc test, using *B. marajoensis* venom as the control/reference group. * *p* < 0.02 and **** *p* < 0.0001 when compared to *B. marajoensis* snake venom.

**Figure 6:**
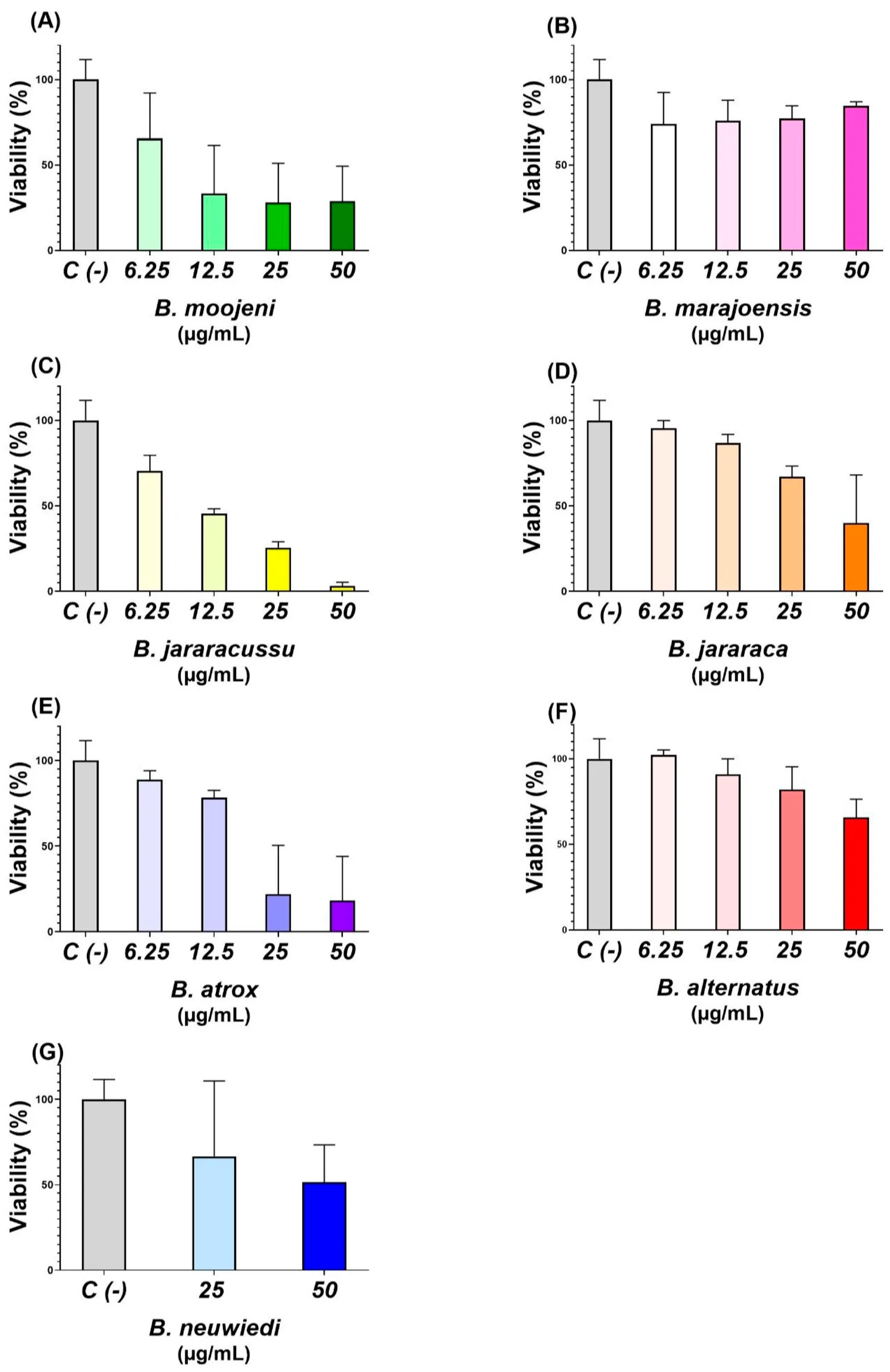
Effects of *Bothrops* spp. venoms on the viability of differentiated C2C12 myotubes. Cells were exposed to venoms (6.25–50 µg/mL, 1.25–10 µg/well, in 200 µL) from (**A**) *B. moojeni*, (**B**) *B. marajoensis*, (**C**) *B. jararacussu*, (**D**) *B. jararaca*, (**E**) *B. atrox*, (**F**) *B. alternatus*, and (**G**) *B. neuwiedi*. Cell viability was determined using an ATP-dependent luminescence assay and expressed as a percentage relative to the negative control (C (−), normalized to 100%). Bars represent the mean ± standard deviation (SD) (n = 3). Data analysis and graphical representation were performed using GraphPad Prism version 8.0.1 (GraphPad Software, San Diego, CA, USA).

**Table 1:** Estimated IC_50_ values of differentiated C2C12 myotubes after *Bothrops* venoms exposure.

Species	Preliminary IC_50_ * (µg/mL)
*B. moojeni*	≈12.00
*B. marajoensis*	>50.00
*B. jararacussu*	≈10.99
*B. jararaca*	>50.00
*B. atrox*	≈17.69
*B. alternatus*	>50.00
*B. neuwiedi*	ND

* IC_50_ values are expressed as µg/mL, considering a final assay volume of 200 µL per well. Values exceeding the highest tested concentration are reported as >50 µg/mL, whereas IC_50_ values that could not be reliably estimated are indicated as not determined (ND).

## Data Availability

The original contributions presented in this study are included in the article. Further inquiries can be directed to the corresponding author.

## Abbreviations

ANOVA	Analysis of variance
CBC	Chick biventer cervicis
CRISPs	Cysteine-rich secretory proteins
CTLs	C-type lectins
ELISA	Enzyme-linked immunosorbent assay
IC_50_	Half-maximal inhibitory concentration
LAAOs	L-amino acid oxidases
LC-MS/MS	Liquid Chromatography tandem Mass Spectrometry
LMW	Low Molecular Weight
MeCN	Acetonitrile
NaOH	Sodium hydroxide
PDE	Phosphodiesterase
PLA_2_	Phospholipases A_2_
PND	Phrenic nerve-diaphragm
RP-FPLC	Reversed-phase protein liquid chromatography
SD	Standard deviation
SDS-PAGE	Sodium Dodecyl Sulfate–Polyacrylamide Gel Electrophoresis
SVMPs	Snake venom metalloproteinases
SVSPs	Snake venom serine proteinases
TFA	Trifluoroacetic acid
TLBm	Thrombin-like serine protease from *Bothrops marajoensis*
ULMW	Ultra-Low Molecular Weight
